# Shape-preserving properties of a new family of generalized Bernstein operators

**DOI:** 10.1186/s13660-018-1821-9

**Published:** 2018-09-14

**Authors:** Qing-Bo Cai, Xiao-Wei Xu

**Affiliations:** 1 School of Mathematics and Computer Science, Quanzhou Normal University, Quanzhou, China; 2 School of Computer and Data Engineering, Ningbo Institute of Technology, Zhejiang University, Ningbo, China

**Keywords:** 65D17, 41A10, 41A25, Bernstein operators, *q*-integers, Shape-preserving, Basis function, Monotonicity

## Abstract

In this paper, we introduce a new family of generalized Bernstein operators based on *q* integers, called $(\alpha,q)$-Bernstein operators, denoted by $T_{n,q,\alpha}(f)$. We investigate a Kovovkin-type approximation theorem, and obtain the rate of convergence of $T_{n,q,\alpha}(f)$ to any continuous functions *f*. The main results are the identification of several shape-preserving properties of these operators, including their monotonicity- and convexity-preserving properties with respect to $f(x)$. We also obtain the monotonicity with *n* and *q* of $T_{n,q,\alpha}(f)$.

## Introduction

A generalization of Bernstein polynomials based on *q*-integers was proposed by Lupaş in 1987 in [1]. However, the Lupaş *q*-Bernstein operators are rational functions rather than polynomials. In 1997, Phillips [2] proposed the Phillips *q*-Bernstein polynomials, and for decades thereafter the application of *q* integers in positive linear operators became a hot topic in approximation theory, such as generalized *q*-Bernstein polynomials [3–6], Durrmeyer-type *q*-Bernstein operators [7–9], Kantorovich-type *q*-Bernstein operators [10–13], etc. As we know, *q* integers play important roles not only in approximation theory, but also in CAGD. Based on the Phillips *q*-Bernstein polynomials [2], which are generalizations of Bernstein polynomials, generalized Bézier curves and surfaces were introduced in [14–16]. In [14], Oruç and Phillips constructed *q*-Bézier curves using the basis functions of Phillips *q*-Bernstein polynomials. Dişibüyük and Oruç [15, 16] defined the *q* generalization of rational Bernstein–Bézier curves and tensor product *q*-Bernstein–Bézier surfaces. Moreover, Simeonov *et al.* [17] introduced a new variant of the blossom, the *q* blossom, which is specifically adapted to developing identities and algorithms for *q*-Bernstein bases and *q*-Bézier curves. In 2014, Han *et al.* [18] proposed a generalization of *q*-analog Bézier curves with one shape parameter, and established degree evaluation and de Casteljau algorithms and some other properties. In 2016, Han *et al.* [19] introduced a new generalization of weighted rational Bernstein–Bézier curves based on *q* integers, and investigated the generalized rational Bézier curve from a geometric point of view, obtaining degree evaluation and de Casteljau algorithms, etc.

Recently, Chen *et al.* [20] introduced a new family of *α*-Bernstein operators, and investigated some approximation properties, such as the rate of convergence, Voronovskaja-type asymptotic formulas, etc. They also obtained the monotonic and convex properties. For $f(x)\in [0,1]$, $n\in \mathbb{N}$, and any fixed real *α*, the *α*-Bernstein operators they introduced are defined as

1 $$\begin{aligned} T_{n,\alpha }=\sum_{i=0}^{n}f_{i}p_{n,i}^{(\alpha )}(x), \end{aligned}$$

where $f_{i}=f ( \frac{i}{n} ) $. For $i=0,1,\ldots,n$, the *α*-Bernstein polynomial $p_{n,i}^{\alpha }(x)$ of degree *n* is defined by $p_{1,0}^{(\alpha )}(x)=1-x$, $p_{1,1}^{(\alpha )}(x)=x$ and

2 $$\begin{array}{rcl} p_{n,i}^{\left(\alpha \right)}(x) & = & \left[\left(\begin{array}{c} n-2\\ i \end{array}\right)(1-\alpha )x+\left(\begin{array}{c} n-2\\ i-2 \end{array}\right)(1-\alpha )(1-x)+\left(\begin{array}{c} n\\ i \end{array}\right)\alpha x(1-x)\right]\\ & & \times x^{i-1}{\left(1-x\right)}^{n-1-i}, \end{array}$$

where $n\geq 2$.

Motivated by above research, in this paper we propose the *q* analogue of *α*-Bernstein operators, called $(\alpha , q)$-Bernstein operators, which are defined as

3 $$ T_{n,q,\alpha }(f;x)=\sum_{i=0}^{n}f_{i}p_{n,q,i}^{(\alpha )}(x), $$

where $q\in (0,1]$, $f_{i}=f ( \frac{[i]_{q}}{[n]_{q}} ) $, $i=0,1,2,\ldots,n$, $p_{1,q,0}^{(\alpha )}(x)=1-x$, $p_{1,q,1}^{(\alpha )}(x)=x$, and

4 $$\begin{array}{rcl} p_{n,q,i}^{\left(\alpha \right)}(x) & = & ({\left[\begin{array}{c} n-2\\ i \end{array}\right]}_{q}(1-\alpha )x+{\left[\begin{array}{c} n-2\\ i-2 \end{array}\right]}_{q}(1-\alpha )q^{n-i-2}\left(1-q^{n-i-1}x\right)\\ & & +{\left[\begin{array}{c} n\\ i \end{array}\right]}_{q}\alpha x\left(1-q^{n-i-1}x\right))x^{i-1}{\left(1-x\right)}_{q}^{n-i-1}\;(n\geq 2). \end{array}$$

By simple computations, we can also express the $(\alpha , q)$ operators (3) as

5 $$\begin{array}{c} T_{n,q,\alpha }(f;x)\\ \;=(1-\alpha )\sum_{i=0}^{n-1}g_{i}{\left[\begin{array}{c} n-1\\ i \end{array}\right]}_{q}x^{i}{\left(1-x\right)}_{q}^{n-1-i}+\alpha \sum_{i=0}^{n}f_{i}{\left[\begin{array}{c} n\\ i \end{array}\right]}_{q}x^{i}{\left(1-x\right)}_{q}^{n-i}, \end{array}$$

where

6 $$\begin{aligned} g_{i}= \biggl( 1-\frac{q^{n-1-i}[i]_{q}}{[n-1]_{q}} \biggr) f_{i}+ \frac{q ^{n-1-i}[i]_{q}}{[n-1]_{q}}f_{i+1}. \end{aligned}$$

Here, we mention some definitions based on *q* integers, the details of which can be found in [21, 22]. For any fixed real number $0< q\leq 1$ and each non-negative integer *k*, we denote *q*-integers by $[k]_{q}$, where

$$ [k]_{q}:= \textstyle\begin{cases} \frac{1-q^{k}}{1-q}, &q\neq 1, \\ k, &q=1. \end{cases} $$

Also, *q*-factorial and *q*-binomial coefficients are defined as follows:

$$\begin{array}{c} {\left[k\right]}_{q}!:=\left\{ \begin{array}{cc} {\left[k\right]}_{q}{\left[k-1\right]}_{q}\cdots {\left[1\right]}_{q}, & k=1,2,\ldots ,\\ 1, & k=0, \end{array}\right.\\ {\left[\begin{array}{c} n\\ k \end{array}\right]}_{q}:=\frac{{\left[n\right]}_{q}!}{{\left[k\right]}_{q}!{\left[n-k\right]}_{q}!}\;(n\geq k\geq 0). \end{array}$$

The *q*-analog of $(1+x)^{n}$ is defined by $(1+x)_{q}^{n}:=\prod_{s=0}^{n-1} ( 1+q^{s}x ) $. The *q* derivative and *q* derivative of the product are defined as $D_{q}f(x):=\frac{d_{q}f(x)}{d _{q}x}=\frac{f(qx)-f(x)}{(q-1)x} $ and $D_{q}(f(x)g(x)):=f(qx)D_{q}g(x)+g(x)D _{q}f(x)$, respectively. We also have $D_{q}x^{n}=[n]_{q}x^{n-1}$ and $D_{q}(1-x)_{q}^{n}=-[n]_{q}(1-qx)_{q}^{n-1}$.

The rest of this paper is organized as follows. In the next section, we give some basic properties of the operators $T_{n,q,\alpha }(f)$, such as the moments and central moments for proving the convergence theorems, the forward difference form of $T_{n,q,\alpha }(f)$ for proving shape-preserving properties, etc. In Sect. 3, we obtain the convergence property and the rate of convergence theorem. In Sect. 4, we investigate some shape-preserving properties, such as monotonicity- and convexity-preserving properties with respect to $f(x)$, and also we study the monotonicity with *n* and *q* of $T_{n,q,\alpha }(f)$.

## Auxiliary results

For proving the main results, we require the following lemmas.

### Lemma 2.1

*We have the following equalities*:

7 $$\begin{aligned} T_{n,q,\alpha }(1;x)=1, \quad\quad T_{n,q,\alpha }(t;x)=x. \end{aligned}$$

### Proof

By (5), we have

$$\begin{array}{rcl} T_{n,q,\alpha }(1;x) & = & (1-\alpha )\sum_{i=0}^{n-1}{\left[\begin{array}{c} n-1\\ i \end{array}\right]}_{q}x^{i}{\left(1-x\right)}_{q}^{n-1-i}+\alpha \sum_{i=0}^{n}{\left[\begin{array}{c} n\\ i \end{array}\right]}_{q}x^{i}{\left(1-x\right)}_{q}^{n-i}\\ & = & 1. \end{array}$$

However,

$$\begin{array}{c} T_{n,q,\alpha }(t;x)\\ \;=(1-\alpha )\sum_{i=0}^{n-1}\left[\left(1-\frac{q^{n-1-i}{\left[i\right]}_{q}}{{\left[n-1\right]}_{q}}\right)\frac{{\left[i\right]}_{q}}{{\left[n\right]}_{q}}+\frac{q^{n-1-i}{\left[i\right]}_{q}}{{\left[n-1\right]}_{q}}\frac{{\left[i+1\right]}_{q}}{{\left[n\right]}_{q}}\right]{\left[\begin{array}{c} n-1\\ i \end{array}\right]}_{q}x^{i}{\left(1-x\right)}_{q}^{n-1-i}\\ \;\;+\alpha \sum_{i=0}^{n}\frac{{\left[i\right]}_{q}}{{\left[n\right]}_{q}}{\left[\begin{array}{c} n\\ i \end{array}\right]}_{q}x^{i}{\left(1-x\right)}_{q}^{n-i}\\ \;=(1-\alpha )\sum_{i=0}^{n-1}\frac{{\left[i\right]}_{q}}{{\left[n-1\right]}_{q}}{\left[\begin{array}{c} n-1\\ i \end{array}\right]}_{q}x^{i}{\left(1-x\right)}_{q}^{n-1-i}+\alpha \sum_{i=0}^{n}\frac{{\left[i\right]}_{q}}{{\left[n\right]}_{q}}{\left[\begin{array}{c} n\\ i \end{array}\right]}_{q}x^{i}{\left(1-x\right)}_{q}^{n-i}\\ \;=(1-\alpha )x+\alpha x=x. \end{array}$$

Lemma 2.1 is proved. □

### Remark 2.2

From Lemma 2.1, we know that the $(\alpha , q)$-Bernstein operators $T_{n,q,\alpha }(f;x)$ reproduce linear functions; that is,

$$\begin{aligned} T_{n,q,\alpha }(at+b;x)=ax+b, \end{aligned}$$

for all real numbers *a* and *b*.

We immediately obtain Lemma 2.3 from (5) and Lemma 2.1.

### Lemma 2.3

*For all functions* *f* *and* *g* *defined in* $[0,1]$, $x\in [0,1]$, *real numbers* *λ*, *μ* *defined in* $[0,1]$, *and* $q\in (0,1]$, *the following statements hold true*.

(i) *Endpoint interpolation*: $T_{n,q,\alpha }(f;0)=f(0)$ *and* $T_{n,q,\alpha }(f;1)=f(1)$.
(ii) *Linearity*: $T_{n,q,\alpha }(\lambda f+\mu g;x)= \lambda T_{n,q,\alpha }(f;x)+\mu T_{n,q,\alpha }(g;x)$.
(iii) *Non*-*negative*: *For* $0\leq \alpha \leq 1$ *and* $0< q<1$, *if* *f* *is non*-*negative on* $[0,1]$, *so is* $(\alpha , q)$-*Bernstein operators* $T_{n,q,\alpha }(f;x)$.
(iv) *Monotone*: *For fixed* $0\leq \alpha \leq 1$ *and* $0< q<1$, *if* $f\geq g$, *then* $T_{n,q,\alpha }(f;x)\geq T_{n,q,\alpha }(g;x)$.

### Lemma 2.4

(i) *The* $(\alpha , q)$-*Bernstein operators may be expressed in the form*
  8 $$T_{n,q,\alpha }(f;x)=\sum_{r=0}^{n}\left((1-\alpha ){\left[\begin{array}{c} n-1\\ r \end{array}\right]}_{q}{△}_{q}^{r}g_{0}+\alpha {\left[\begin{array}{c} n\\ r \end{array}\right]}_{q}{△}_{q}^{r}f_{0}\right)x^{r},$$
  *where* ${\left[\begin{array}{c} n-1\\ n \end{array}\right]}_{q}=0$, $\triangle_{q}^{r}f_{j}=\triangle_{q}^{r-1}f_{j+1}-q ^{r-1}\triangle_{q}^{r-1}f_{j}$, $r\geq 1$, *with* $\triangle_{q}^{0}f _{j}=f_{j}=f ( \frac{[j]_{q}}{[n]_{q}} ) $.
(ii) *The higher*-*order forward difference of* $g_{i}$ *may be expressed in the form*
  9 $$\begin{aligned} \triangle_{q}^{r}g_{i}= \biggl( 1- \frac{q^{n-i-1}[i]_{q}}{[n-1]_{q}} \biggr) \triangle_{q}^{r}f_{i}+ \frac{q^{n-i-1-r}[i+r]_{q}}{[n-1]_{q}}\triangle _{q}^{r}f_{i+1}, \end{aligned}$$
  *where* $\triangle_{q}^{0}g_{i}=g_{i}$, *which is defined in* (6).

### Proof

We can obtain (8) easily by [2]. Next, in order to prove (9), we use induction on *r*. It is clear that (9) holds for $r=0$. Let us assume that (9) holds for some $r=k\geq 0$. For $r=k+1$, we have

$$\begin{aligned}& \triangle_{q}^{k+1}g_{i} \\& \quad = \triangle_{q}^{k}g_{i+1}-q^{k} \triangle_{q}^{k}g_{i} \\& \quad = \biggl( 1-\frac{q^{n-i-2}[i+1]_{q}}{[n-1]_{q}} \biggr) \triangle_{q} ^{k}f_{i+1}+\frac{q^{n-i-2-k}[i+k+1]_{q}}{[n-1]_{q}}\triangle_{q}^{k}f _{i+2} \\& \quad \quad {} -q^{k} \biggl[ \biggl( 1-\frac{q^{n-i-1}[i]_{q}}{[n-1]_{q}} \biggr) \triangle_{q}^{k}f_{i}+\frac{q^{n-i-k-1}[i+k]_{q}}{[n-1]_{q}} \triangle _{q}^{k}f_{i+1} \biggr] \\& \quad = \biggl[ 1-\frac{q^{n-i-2} ( 1+q[i]_{q} ) }{[n-1]_{q}} \biggr] \triangle_{q}^{k}f_{i+1}- \biggl( 1-\frac{q^{n-i-1}[i]_{q}}{[n-1]_{q}} \biggr) q ^{k}\triangle_{q}^{k}f_{i} \\& \quad \quad {} -\frac{q^{n-i-1}[i+k]_{q}}{[n-1]_{q}}\triangle_{q}^{k}f_{i+1}+ \frac{q ^{n-i-2-k}[i+k]_{q}}{[n-1]_{q}}\triangle_{q}^{k}f_{i+2} \\& \quad = \biggl( 1-\frac{q^{n-i-1}[i]_{q}}{[n-1]_{q}} \biggr) \triangle_{q}^{k+1}f _{i}-\frac{q^{n-i-2}}{[n-1]_{q}}\triangle_{q}^{k}f_{i+1}- \frac{q^{n-i-1}[i+k]_{q}}{[n-1]_{q}}\triangle_{q}^{k}f_{i+1} \\& \quad \quad {} +\frac{q^{n-i-2-k}[i+k+1]_{q}}{[n-1]_{q}}\triangle_{q}^{k}f_{i+2} \\& \quad = \biggl( 1-\frac{q^{n-i-1}[i]_{q}}{[n-1]_{q}} \biggr) \triangle_{q}^{k+1}f _{i}-\frac{q^{n-i-2}[i+k+1]_{q}}{[n-1]_{q}}\triangle_{q}^{k}f_{i+1}+ \frac{q ^{n-i-1-k}[i+k+1]_{q}}{[n-1]_{q}}\triangle_{q}^{k}f_{i+2} \\& \quad = \biggl( 1-\frac{q^{n-i-1}[i]_{q}}{[n-1]_{q}} \biggr) \triangle_{q}^{k+1}f _{i}+\frac{q^{n-i-k-2}[i+k+1]_{q}}{[n-1]_{q}} \bigl( \triangle_{q}^{k}f _{i+2}-q^{k}\triangle_{q}^{k}f_{i+1} \bigr) \\& \quad = \biggl( 1-\frac{q^{n-i-1}[i]_{q}}{[n-1]_{q}} \biggr) \triangle_{q}^{k+1}f _{i}+\frac{q^{n-i-k-2}[i+k+1]_{q}}{[n-1]_{q}}\triangle_{q}^{k+1}f_{i+1}. \end{aligned}$$

This shows that (9) holds when *k* is replaced by $k+1$, and this completes the proof of Lemma 2.4. □

Since $f [ \frac{[j]_{q}}{[n]_{q}},\frac{[j+1]_{q}}{[n]_{q}},\ldots, \frac{[j+k]_{q}}{[n]_{q}} ] =\frac{[n]_{q}^{k}\triangle_{q}^{k}f _{j}}{q^{\frac{k(2j+k-1)}{2}}[k]_{q}!}=\frac{f^{(k)}(\xi )}{k!}$, where $\xi \in ( \frac{[j]_{q}}{[n]_{q}},\frac{[j+k]_{q}}{[n]_{q}} ) $, the *q* differences of the monomial $x^{k}$ of order greater than *k* are zero. We see from Lemma 2.4 that, for all $n\geq k$, $T_{n,q,\alpha } ( t^{k};x ) $ is a polynomial of degree *k*. Actually, the $(\alpha , q)$-Bernstein operators are degree-reducing on polynomials; that is, if *f* is a polynomial of degree *m*, and then $T_{n,q,\alpha }(f)$ is a polynomial of degree $\leq \min\{m,n\}$. In particular, we have the following results.

### Lemma 2.5

*Letting* $f(t)=t^{k}$, $n-1\geq k\geq 2$, *we have*

$$\begin{aligned} T_{n,q,\alpha }\bigl(t^{k};x\bigr)=a_{k}x^{k}+a_{k-1}x^{k-1}+ \cdots+a_{1}x+a_{0}, \end{aligned}$$

*where* $a_{k}=\frac{q^{\frac{k(k-1)}{2}}[n-2]_{q}!}{[n-k]_{q}![n]_{q} ^{k}} \{ (1-\alpha )[n-k]_{q}[n-1+k]_{q}+\alpha [n]_{q}[n-1]_{q} \} $.

### Proof

Indeed, from (9) and $\triangle_{q}^{k}f_{j}=\frac{q^{ \frac{k(2j+k-1)}{2}}[k]_{q}!f^{(k)}(\xi )}{k![n]_{q}^{k}}$, we have

$$\begin{aligned} \triangle_{q}^{k}g_{0}=\triangle_{q}^{k}f_{0}+ \frac{q^{n-1-k}[k]_{q}}{[n-1]_{q}}\triangle_{q}^{k}f_{1},\quad\quad \triangle_{q}^{k}f_{0}=\frac{q^{\frac{k(k-1)}{2}}[k]_{q}!}{[n]_{q} ^{k}},\quad\quad \triangle_{q}^{k}f_{1}=\frac{q^{\frac{k(k+1)}{2}}[k]_{q}!}{[n]_{q} ^{k}}. \end{aligned}$$

Thus, we obtain

$$\begin{aligned} \triangle_{q}^{k}g_{0}= \biggl( 1+ \frac{q^{n-1}[k]_{q}}{[n-1]_{q}} \biggr) \frac{q ^{\frac{k(k-1)}{2}}[k]_{q}!}{[n]_{q}^{k}}= \frac{[n-1+k]_{q}}{[n-1]_{q}} \frac{q^{\frac{k(k-1)}{2}}[k]_{q}!}{[n]_{q} ^{k}}. \end{aligned}$$

Hence, using (8), we have

$$a_{k}=\left[(1-\alpha ){\left[\begin{array}{c} n-1\\ k \end{array}\right]}_{q}\frac{{\left[n-1+k\right]}_{q}}{{\left[n-1\right]}_{q}}+\alpha {\left[\begin{array}{c} n\\ k \end{array}\right]}_{q}\right]\frac{q^{\frac{k(k-1)}{2}}{\left[k\right]}_{q}!}{{\left[n\right]}_{q}^{k}}.$$

We then obtain the proof of Lemma 2.5 by simple computations. □

### Lemma 2.6

*The following equalities hold true*:

10 $$\begin{aligned}& T_{n,q,\alpha } \bigl( t^{2};x \bigr) =x^{2}+ \frac{x(1-x)}{[n]_{q}}+\frac{(1- \alpha )q^{n-1}[2]_{q}x(1-x)}{[n]_{q}^{2}}, \end{aligned}$$

11 $$\begin{aligned}& T_{n,q,\alpha } \bigl( (t-x)^{2};x \bigr) =\frac{x(1-x)}{[n]_{q}}+ \frac{(1- \alpha )q^{n-1}[2]_{q}x(1-x)}{[n]_{q}^{2}}. \end{aligned}$$

### Proof

For $f(t)=t^{2}$, we have $\triangle_{q}^{0}f_{0}=f_{0}=0$, $\triangle_{q}^{1}f_{0}=f_{1}-f_{0}=\frac{1}{[n]_{q}^{2}}$, $\triangle_{q}^{1}f_{1}=f_{2}-f_{1}=\frac{2q+q^{2}}{[n]_{q}^{2}}$, $\triangle_{q}^{2}f_{0}=\triangle_{q}^{1}f_{1}-q\triangle_{q}^{1}f _{0}=f_{2}-[2]_{q}f_{1}+qf_{0}=\frac{q[2]_{q}}{[n]_{q}^{2}}$, and $\triangle_{q}^{2}f_{1}=f_{3}-[2]_{q}f_{2}+qf_{1}=\frac{q^{3}+q^{4}}{[n]_{q} ^{2}}$. By (9), we have $\triangle_{q}^{0}g_{0}=0$, and

$$\begin{aligned}& \triangle_{q}^{1}g_{0}=\triangle_{q}^{1}f_{0}+ \frac{q^{n-2}}{[n-1]_{q}}\triangle_{q}^{1}f_{1}= \frac{1}{[n]_{q}^{2}}+\frac{2q ^{n-1}+q^{n}}{[n-1]_{q}[n]_{q}^{2}}, \\& \triangle_{q}^{2}g_{0}=\triangle_{q}^{2}f_{0}+ \frac{q^{n-3}[2]_{q}}{[n-1]_{q}}\triangle_{q}^{2}f_{1}= \frac{q[2]_{q}}{[n]_{q} ^{2}}+\frac{[2]_{q} ( q^{n}+q^{n+1} ) }{[n-1]_{q}[n]_{q}^{2}}. \end{aligned}$$

From (8), we have

$$\begin{aligned}& T_{n,q,\alpha } \bigl( t^{2};x \bigr) \\& \quad = (1-\alpha )\triangle_{q}^{0}g_{0}+\alpha \triangle_{q}^{0}f_{0}+ \bigl[ (1-\alpha )[n-1]_{q}\triangle_{q}^{1}g_{0}+ \alpha [n]_{q} \triangle_{q}^{1}f_{0} \bigr] x \\& \quad \quad {} + \biggl[ (1-\alpha )\frac{[n-1]_{q}[n-2]_{q}}{[2]_{q}}\triangle_{q} ^{2}g_{0}+\alpha \frac{[n]_{q}[n-1]_{q}}{[2]_{q}}\triangle_{q}^{2}f _{0} \biggr] x^{2} \\& \quad = \biggl[ \frac{(1-\alpha )[n-1]_{q}}{[n]_{q}^{2}}+\frac{(1-\alpha ) ( 2q^{n-1}+q^{n} ) }{[n]_{q}^{2}}+\frac{\alpha }{[n]_{q}} \biggr] x \\& \quad \quad {} + \biggl[ \frac{(1-\alpha )q[n-1]_{q}[n-2]_{q}}{[n]_{q}^{2}}+\frac{(1- \alpha )[n-2]_{q} ( q^{n}+q^{n+1} ) }{[n]_{q}^{2}}+\frac{ \alpha q[n-1]_{q}}{[n]_{q}} \biggr] x^{2} \\& \quad = \frac{[n]_{q}+(1-\alpha )q^{n-1}[2]_{q}}{[n]_{q}^{2}}x+ \biggl( 1- \frac{1}{[n]_{q}}-\frac{(1-\alpha )q^{n-1}[2]_{q}}{[n]_{q}^{2}} \biggr) x ^{2} \\& \quad = x^{2}+\frac{x(1-x)}{[n]_{q}}+\frac{(1-\alpha )q^{n-1}[2]_{q}x(1-x)}{[n]_{q} ^{2}}. \end{aligned}$$

Hence, (10) is proved. Finally, using Lemma 2.1, we obtain

$$ T_{n,q,\alpha } \bigl( (t-x)^{2};x \bigr) =T_{n,q,\alpha } \bigl( t^{2};x \bigr) -2xT _{n,q,\alpha }(t;x)+x^{2}T_{n,q,\alpha }(1;x)=T_{n,q,\alpha } \bigl( t ^{2};x \bigr) -x^{2}. $$

Then (11) is proved by (10). This completes the proof of Lemma 2.6. □

## Convergence properties

We now state the well-known Bohman–Korovkin theorem, followed by a proof based on that given by Cheney [23].

### Theorem 3.1

*Let* $\{L_{n}\}$ *denote a sequence of monotone linear operators that map a function* $f\in C[a,b]$ *to a function* $L_{n}f\in C[a,b]$, *and let* $L_{n}f\rightarrow f$ *uniformly on* $[a,b]$ *for* $f=1, t$ *and* $t^{2}$. *Then* $L_{n}f\rightarrow f$ *uniformly on* $[a,b]$ *for all* $f\in C[a,b]$.

Theorem 3.1 leads to the following theorem on the convergence of $(\alpha , q)$-Bernstein operators.

### Theorem 3.2

*Let* $q:=\{q_{n}\}$ *denote a sequence such that* $q_{n}\in (0,1)$ *and* $\lim_{n\rightarrow \infty }q_{n}=1$. *Then*, *for any* $f\in C[0,1]$ *and* $\alpha \in [0,1]$, $T_{n,q,\alpha }(f;x)$ *converges uniformly to* $f(x)$ *on* $[0,1]$.

### Proof

From Lemma 2.1, we see that $T_{n,q,\alpha }(f;x)=f(x)$ for $f(t)=1$ and $f(t)=t$. Since $\lim_{n\rightarrow \infty }q_{n}=1$, we see from (10) that $T_{n,q,\alpha }(f;x)$ converges uniformly to $f(x)$ for $f(t)=t^{2}$ as $n\rightarrow \infty $. It also follows that $T_{n,q,\alpha }$ is a monotone operator by Lemma 2.3; the proof is then completed by applying the Bohman–Korovkin theorem 3.1. □

As we know, the space $C{[0,1]}$ of all continuous functions on $[0,1]$ is a Banach space with sup-norm $\Vert f \Vert :=\sup_{x\in [0,1]} \vert f(x) \vert $. Letting $f\in C{[0,1]}$, the Peetre *K* functional is defined by $K_{2}(f;\delta ):=\inf_{g\in C^{2}{[0,1]}} \{ \Vert f-g \Vert +\delta \Vert g'' \Vert \}$, where $\delta >0$ and $C^{2}{[0,1]}:=\{g \in C{[0,1]}: g', g''\in C{[0,1]}\}$. By [24], there exists an absolute constant $C>0$, such that

12 $$ K_{2}(f;\delta )\leq C\omega_{2} ( f;\sqrt{\delta } ) , $$

where $\omega_{2}(f;\delta ):=\sup_{0< h\leq \delta } \sup_{x,x+h,x+2h\in [0,1]} \vert f(x+2h)-2f(x+h)+f(x) \vert $ is the second-order modulus of smoothness of $f\in C{[0,1]}$.

### Theorem 3.3

*For* $f\in C{[0,1]}$, $\alpha \in [0,1]$, $q\in (0,1)$, *we have*

$$\begin{aligned} \bigl\vert T_{n,q,\alpha }(f;x)-f(x) \bigr\vert \leq C\omega_{2} \biggl( f;\frac{\sqrt{2[n]_{q}+(1- \alpha )2[2]_{q}q^{n-1}}}{4[n]_{q}} \biggr) , \end{aligned}$$

*where* *C* *is a positive constant*.

### Proof

Letting $g\in C^{2}{[0,1]}$, $x,t\in [0,1]$, by Taylor’s expansion we have

$$\begin{aligned} g(t)=g(x)+g'(x) (t-x)+ \int_{x}^{t}(t-u)g''(u) \,du. \end{aligned}$$

Using Lemma 2.1, we obtain

$$\begin{aligned} T_{n,q,\alpha }(g;x)=g(x)+T_{n,q,\alpha } \biggl( \int_{x}^{t}(t-u)g''(u) \,du;x \biggr) . \end{aligned}$$

Thus, we have

13 $$\begin{aligned} \bigl\vert T_{n,q,\alpha }(g;x)-g(x) \bigr\vert =& \biggl\vert T_{n,q,\alpha } \biggl( \int _{x}^{t}(t-u)g''(u) \,du;x \biggr) \biggr\vert \\ \leq &T_{n,q,\alpha } \biggl( \biggl\vert \int_{x}^{t}(t-u) \bigl\vert g''(u) \bigr\vert \,du \biggr\vert ;x \biggr) \\ \leq &T_{n,q,\alpha } \bigl( (t-x)^{2};x \bigr) \bigl\Vert g'' \bigr\Vert \\ \leq &\frac{[n]_{q}+(1-\alpha )q^{n-1}[2]_{q}}{4[n]_{q}^{2}} \bigl\Vert g'' \bigr\Vert . \end{aligned}$$

However, using Lemma 2.1, we have

14 $$\begin{aligned} \bigl\vert T_{n,q,\alpha }(f;x) \bigr\vert \leq \Vert f \Vert . \end{aligned}$$

Now, (13) and (14) imply

$$\begin{aligned} \bigl\vert T_{n,q,\alpha }(f;x)-f(x) \bigr\vert \leq & \bigl\vert T_{n,q,\alpha }(f-g;x)-(f-g) (x) \bigr\vert + \bigl\vert T_{n,q,\alpha }(g;x)-g(x) \bigr\vert \\ \leq &2 \Vert f-g \Vert + \frac{[n]_{q}+(1-\alpha )q^{n-1}[2]_{q}}{4[n]_{q}^{2}} \bigl\Vert g'' \bigr\Vert . \end{aligned}$$

Hence, taking the infimum on the right-hand side over all $g\in C^{2} {[0,1]}$, we obtain

$$\begin{aligned} \bigl\vert T_{n,q,\alpha }(f;x)-f(x) \bigr\vert \leq 2K_{2} \biggl( f;\frac{[n]_{q}+(1- \alpha )q^{n-1}[2]_{q}}{8[n]_{q}^{2}} \biggr) . \end{aligned}$$

By (12), we obtain

$$\begin{aligned} \bigl\vert T_{n,q,\alpha }(f;x)-f(x) \bigr\vert \leq C\omega_{2} \biggl( f;\frac{\sqrt{2[n]_{q}+(1- \alpha )2[2]_{q}q^{n-1}}}{4[n]_{q}} \biggr) , \end{aligned}$$

where *C* is a positive constant. Theorem 3.3 is proved. □

### Remark 3.4

Letting $q:=\{q_{n}\}$ denote a sequence such that $q_{n}\in (0,1)$ and $\lim_{n\rightarrow \infty }q_{n}=1$, we know that, under the conditions of theorem 3.3, the convergence rate of the operators $T_{n,q, \alpha }(f)$ to *f* is $1/\sqrt{[n]_{q}}$ as $n\rightarrow \infty $. This convergence rate can be improved depending on the choice of *q*, at least as fast as $1/\sqrt{n}$.

### Example 3.5

**Figure 1:**
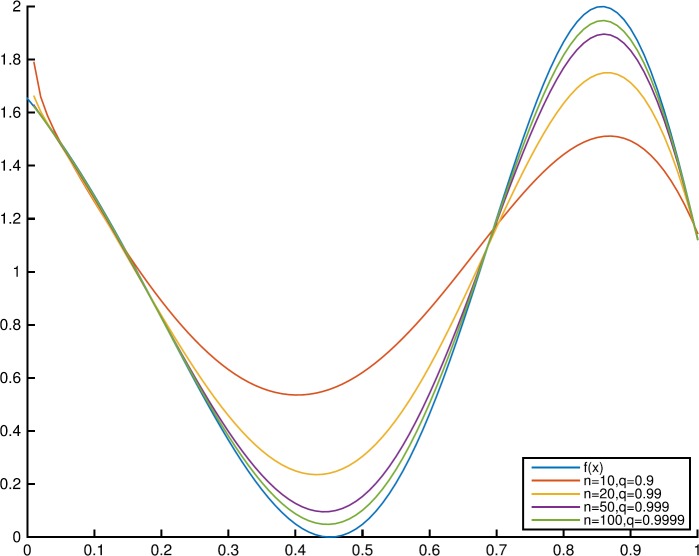
Convergence of $T_{n,q,\alpha }(f;x)$ to $f(x)$ for fixed $\alpha =0.9$

**Figure 2:**
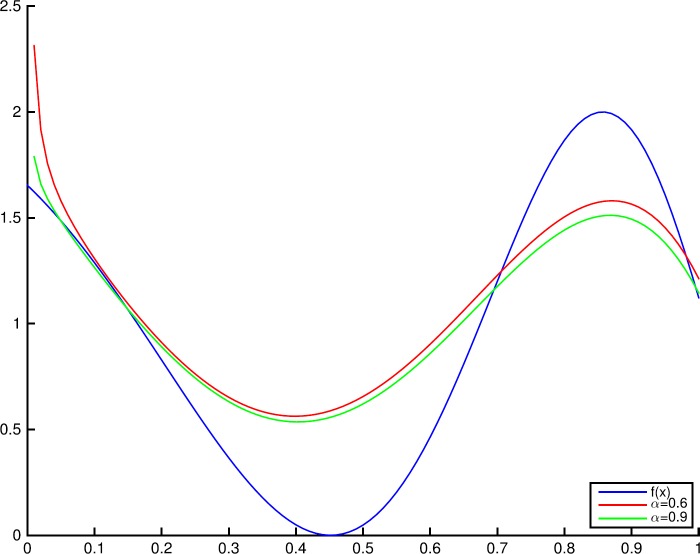
Convergence of $T_{n,q,\alpha }(f;x)$ to $f(x)$ for fixed $q=0.9$

Letting $f(x) = 1 - \cos(4e^{x})$, the graphs of $f(x)$ and $T_{n,q,0.9}(f;x)$ with different values of *n* and *q* are shown in Fig. 1. Figure 2 shows the graphs of $f(x)$ and $T_{10,0.9,\alpha }(f;x)$ with $\alpha =0.6$ and $\alpha =0.9$.

## Shape-preserving properties

The $(\alpha , q)$-Bernstein operators $T_{n,q,\alpha }(f;x)$ have a monotonicity-preserving property.

### Theorem 4.1

*Let* $f\in C{[0,1]}$. *If* *f* *is a monotonically increasing or monotonically decreasing function on* $[0,1]$, *so are all its* $(\alpha , q)$-*Bernstein operators for fixed* $q\in (0,1)$ *and* $\alpha \in [0,1]$.

### Proof

From (5), we have

$$T_{n+1,q,\alpha }(f;x)=(1-\alpha )\sum_{i=0}^{n}g_{i}{\left[\begin{array}{c} n\\ i \end{array}\right]}_{q}x^{i}{\left(1-x\right)}_{q}^{n-i}+\alpha \sum_{i=0}^{n+1}f_{i}{\left[\begin{array}{c} n+1\\ i \end{array}\right]}_{q}x^{i}{\left(1-x\right)}_{q}^{n+1-i},$$

where $f_{i}=\frac{[i]_{q}}{[n+1]_{q}}$, $g_{i}= ( 1- \frac{q^{n-i}[i]_{q}}{[n]_{q}} ) f_{i}+ \frac{q^{n-i}[i]_{q}}{[n]_{q}}f_{i+1}$. Then the *q* derivative of $T_{n+1,q,\alpha }(f;x)$ is

$$\begin{array}{c} D_{q}\left[T_{n+1,q,\alpha }(f;x)\right]\\ \;=(1-\alpha )\sum_{i=0}^{n}g_{i}{\left[\begin{array}{c} n\\ i \end{array}\right]}_{q}D_{q}\left[x^{i}{\left(1-x\right)}_{q}^{n-i}\right]+\alpha \sum_{i=0}^{n+1}f_{i}{\left[\begin{array}{c} n+1\\ i \end{array}\right]}_{q}D_{q}\left[x^{i}{\left(1-x\right)}_{q}^{n+1-i}\right], \end{array}$$

and we denote the first and second parts of the right-hand side of the last equation by $\Lambda_{1}$ and $\Lambda_{2}$, respectively. We then have

$$\begin{array}{c} \Lambda_{1}\\ \;=(1-\alpha )\sum_{i=0}^{n}g_{i}{\left[\begin{array}{c} n\\ i \end{array}\right]}_{q}\left[{\left[i\right]}_{q}x^{i-1}{\left(1-qx\right)}_{q}^{n-i}-{\left[n-i\right]}_{q}x^{i}{\left(1-qx\right)}_{q}^{n-i-1}\right]\\ \;=(1-\alpha ){\left[n\right]}_{q}\left[\sum_{i=1}^{n}g_{i}{\left[\begin{array}{c} n-1\\ i-1 \end{array}\right]}_{q}x^{i-1}{\left(1-qx\right)}_{q}^{n-i}-\sum_{i=0}^{n-1}g_{i}{\left[\begin{array}{c} n-1\\ i \end{array}\right]}_{q}x^{i}{\left(1-qx\right)}_{q}^{n-i-1}\right]\\ \;=(1-\alpha ){\left[n\right]}_{q}\sum_{i=0}^{n-1}{\left[\begin{array}{c} n-1\\ i \end{array}\right]}_{q}x^{i}{\left(1-qx\right)}_{q}^{n-i-1}{△}_{q}^{1}g_{i}. \end{array}$$

Using (9), we obtain

$$\begin{aligned} \triangle_{q}^{1}g_{i}= \biggl( 1- \frac{q^{n-i}[i]_{q}}{[n]_{q}} \biggr) \triangle_{q}^{1}f_{i}+ \frac{q^{n-i-1}[i+1]}{[n]_{q}}\triangle_{q} ^{1}f_{i+1}. \end{aligned}$$

Thus, we have

15 $$\begin{array}{rcl} \Lambda_{1} & = & (1-\alpha )\sum_{i=0}^{n-1}\left[\left({\left[n\right]}_{q}-q^{n-i}{\left[i\right]}_{q}\right){△}_{q}^{1}f_{i}+q^{n-i-1}{\left[i+1\right]}_{q}{△}_{q}^{1}f_{i+1}\right]{\left[\begin{array}{c} n-1\\ i \end{array}\right]}_{q}\\ & & \times x^{i}{\left(1-qx\right)}_{q}^{n-i-1}. \end{array}$$

Similarly, we can obtain

16 $$\Lambda_{2}=\alpha {\left[n+1\right]}_{q}\sum_{i=0}^{n}{\left[\begin{array}{c} n\\ i \end{array}\right]}_{q}x^{i}{\left(1-qx\right)}_{q}^{n-i}{△}_{q}^{1}f_{i}.$$

Therefore, by using (15) and (16), the derivative of $( \alpha , q)$-Bernstein operators $T_{n,q,\alpha }(f;x)$ may be expressed in the form

$$\begin{array}{c} D_{q}\left[T_{n,q,\alpha }(f;x)\right]\\ \;=(1-\alpha )\sum_{i=0}^{n-1}\left[\left({\left[n\right]}_{q}-q^{n-i}{\left[i\right]}_{q}\right){△}_{q}^{1}f_{i}+q^{n-i-1}{\left[i+1\right]}_{q}{△}_{q}^{1}f_{i+1}\right]{\left[\begin{array}{c} n-1\\ i \end{array}\right]}_{q}\\ \;\;\times x^{i}{\left(1-qx\right)}_{q}^{n-i-1}+\alpha {\left[n+1\right]}_{q}\sum_{i=0}^{n}{\left[\begin{array}{c} n\\ i \end{array}\right]}_{q}x^{i}{\left(1-qx\right)}_{q}^{n-i}{△}_{q}^{1}f_{i}. \end{array}$$

Since if *f* is monotonically increasing on $[0,1]$, the forward differences $\triangle_{q}^{1}f_{i}$ and $\triangle_{q}^{1}f_{i+1}$ are non-negative, and so is $D_{q} [ T_{n,q,\alpha }(f;x) ] $. Hence, $(\alpha , q)$-Bernstein operators $T_{n,q,\alpha }(f;x)$ are monotonically increasing on $[0,1]$ for fixed $q\in (0,1)$ and $\alpha \in [0,1]$. On the contrary, if *f* is monotonically decreasing on $[0,1]$, then operators $T_{n,q,\alpha }(f;x)$ are monotonically decreasing on $[0,1]$ for fixed $q\in (0,1)$ and $\alpha \in [0,1]$. Theorem 4.1 is proved. □

The $(\alpha , q)$-Bernstein operators $T_{n,q,\alpha }(f;x)$ have a convexity-preserving property

### Theorem 4.2

*Let* $f\in C{[0,1]}$. *If* *f* *is convex on* $[0,1]$, *so are all of its* $( \alpha , q)$-*Bernstein operators* $T_{n,q,\alpha }(f;x)$ *for fixed* $q\in (0,1)$ *and* $\alpha \in [0,1]$.

### Proof

From (5), we obtain

$$\begin{array}{c} T_{n+2,q,\alpha }(f;x)\\ \;=(1-\alpha )\sum_{i=0}^{n+1}g_{i}{\left[\begin{array}{c} n+1\\ i \end{array}\right]}_{q}x^{i}{\left(1-x\right)}_{q}^{n-i+1}+\alpha \sum_{i=0}^{n+2}f_{i}{\left[\begin{array}{c} n+2\\ i \end{array}\right]}_{q}x^{i}{\left(1-x\right)}_{q}^{n+2-i}, \end{array}$$

where $f_{i}=\frac{[i]_{q}}{[n+2]_{q}}$, $g_{i}= ( 1- \frac{q^{n-i+1}[i]_{q}}{[n+1]_{q}} ) f_{i}+ \frac{q^{n-i+1}[i]_{q}}{[n+1]_{q}}f_{i+1}$. The *q*-derivative of $T_{n+2,q,\alpha }(f;x)$ can easily obtained by the proof theorem 4.1, which may be expressed as

$$\begin{array}{rcl} D_{q}\left[T_{n+2,q,\alpha }(f;x)\right] & = & \left(1-\alpha ){\left[n+1\right]}_{q}\sum_{i=0}^{n}{\left[\begin{array}{c} n\\ i \end{array}\right]}_{q}x^{i}{\left(1-qx\right)}_{q}^{n-i}(g_{i+1}-g_{i}\right)\\ & & +\alpha {\left[n+2\right]}_{q}\sum_{i=0}^{n+1}{\left[\begin{array}{c} n+1\\ i \end{array}\right]}_{q}x^{i}{\left(1-qx\right)}_{q}^{n-i+1}(f_{i+1}-f_{i}). \end{array}$$

Then we have

$$\begin{array}{c} D_{q}^{2}\left[T_{n+2,q,\alpha }(f;x)\right]\\ \;=(1-\alpha ){\left[n+1\right]}_{q}\sum_{i=0}^{n}{\left[\begin{array}{c} n\\ i \end{array}\right]}_{q}(g_{i+1}-g_{i})D_{q}\left[x^{i}{\left(1-qx\right)}_{q}^{n-i}\right]\\ \;\;+\alpha {\left[n+2\right]}_{q}\sum_{i=0}^{n+1}{\left[\begin{array}{c} n+1\\ i \end{array}\right]}_{q}(f_{i+1}-f_{i})D_{q}\left[x^{i}{\left(1-qx\right)}_{q}^{n-i-1}\right]. \end{array}$$

By some easy computations, we obtain

$$\begin{array}{rcl} D_{q}^{2}\left[T_{n+2,q,\alpha }(f;x)\right] & = & (1-\alpha ){\left[n+1\right]}_{q}{\left[n\right]}_{q}\sum_{i=0}^{n-1}{\left[\begin{array}{c} n-1\\ i \end{array}\right]}_{q}x^{i}{\left(1-q^{2}x\right)}_{q}^{n-i-1}{△}_{q}^{2}g_{i}\\ & & +\alpha {\left[n+2\right]}_{q}{\left[n+1\right]}_{q}\sum_{i=0}^{n}{\left[\begin{array}{c} n\\ i \end{array}\right]}_{q}x^{i}{\left(1-q^{2}x\right)}_{q}^{n-i}{△}_{q}^{2}f_{i}, \end{array}$$

where $\triangle_{q}^{2}g_{i}= ( 1- \frac{q^{n-i+1}[i]_{q}}{[n+1]_{q}} ) \triangle_{q}^{2}f_{i}+\frac{q ^{n-i-1}[i+2]_{q}}{[n+1]_{q}}\triangle_{q}^{2}f_{i+1}$. By the connection between the second-order *q* differences and convexity, we know that $\triangle_{q}^{2}f_{i}$ and $\triangle_{q}^{2}f_{i+1}$ are all non-negative since *f* is convex on $[0,1]$. Hence, we obtain $D_{q}^{2} [ T_{n+2,q,\alpha }(f;x) ] \geq 0$, and then the convexity-preserving property of $T_{n,q,\alpha }(f;x)$. Theorem 4.2 is proved. □

Next, if $f(x)$ is convex, the $(\alpha , q)$-Bernstein operators $T_{n,q,\alpha }(f;x)$, for *n* fixed, are monotonic in *q*.

### Theorem 4.3

*For* $0< q_{1}\leq q_{2}\leq 1$, $\alpha \in [0,1]$ *and for* $f(x)$ *convex on* $[0,1]$, *then* $T_{n,q_{2},\alpha }(f;x)\leq T_{n,q_{1},\alpha }(f;x)$.

### Proof

In the following main proof of our results, we must introduce a linear polynomial function:

17 $$\begin{aligned} g(x)=\frac{f_{i+1}-f_{i}}{\frac{[i+1]_{q}}{[n]_{q}}- \frac{[i]_{q}}{[n]_{q}}} \biggl( x-\frac{[i]_{q}}{[n]_{q}} \biggr) +f_{i}, \end{aligned}$$

where $\frac{[i]_{q}}{[n]_{q}}\leq x<\frac{[i+1]_{q}}{[n]_{q}}$, $f _{i}=f ( \frac{[i]_{q}}{[n]_{q}} ) $, $i=0,\ldots,n-1$. Then it is straightforward to check that $g_{i}=g ( \frac{[i]_{q}}{[n-1]_{q}} ) $. Since *f* is convex on $[0,1]$, the intrinsic linear polynomial function $g(x)$ must be convex on $[0,1]$ as well. Therefore, by the classical results of *q*-Bernstein operators (see [3]), we note that

18 $$\begin{aligned} T_{n,q,\alpha }(f;x)=(1-\alpha )B_{n-1}^{q}(g;x)+\alpha B_{n}^{q}(f;x). \end{aligned}$$

We have $B_{n-1}^{q_{2}}(g;x)\leq B_{n-1}^{q_{1}}(g;x)$ and $B_{n}^{q_{2}}(f;x)\leq B_{n}^{q_{1}}(f;x)$, and the desired result is obvious. Theorem 4.3 is proved. □

Finally, if $f(x)$ is convex, we give the monotonicity of $(\alpha , q)$-Bernstein operators $T_{n,q,\alpha }(f; x)$ with *n*.

### Theorem 4.4

*If* $f(x)$ *is convex on* $[0,1]$, *for fixed* $q\in (0,1)$ *and* $\alpha \in [0,1]$, *we have*

$$\begin{aligned} T_{n-1,q,\alpha }(f;x)-T_{n,q,\alpha }(f;x)\geq 0\quad (n\geq 2). \end{aligned}$$

### Proof

Combining (17) and (18), and the fact that if *f* and *g* are convex on $[0,1]$, then

$$\begin{aligned} B_{n-2}^{q}(g;x)\geq B_{n-1}^{q}(g;x), \quad\quad B_{n-1}^{q}(f;x)\geq B_{n}^{q}(f;x) \end{aligned}$$

(see [25]). The desired result is obvious. □

### Example 4.5

**Figure 3:**
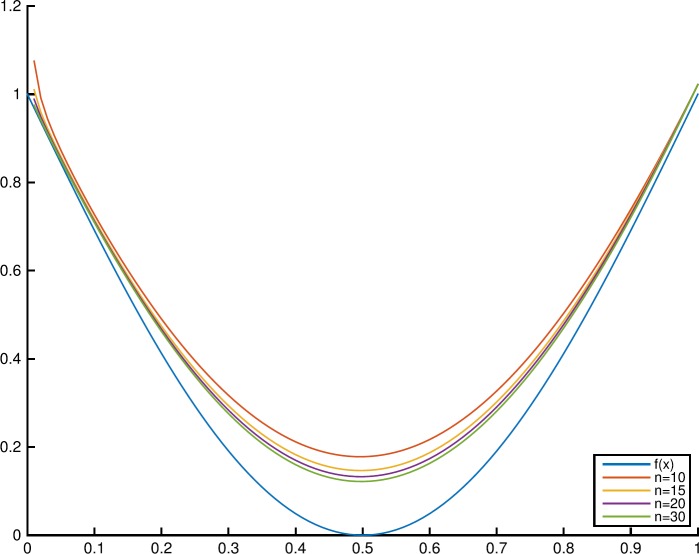
Monotonicity of $T_{n,q,\alpha }(f;x)$ in the parameter *n*

**Figure 4:**
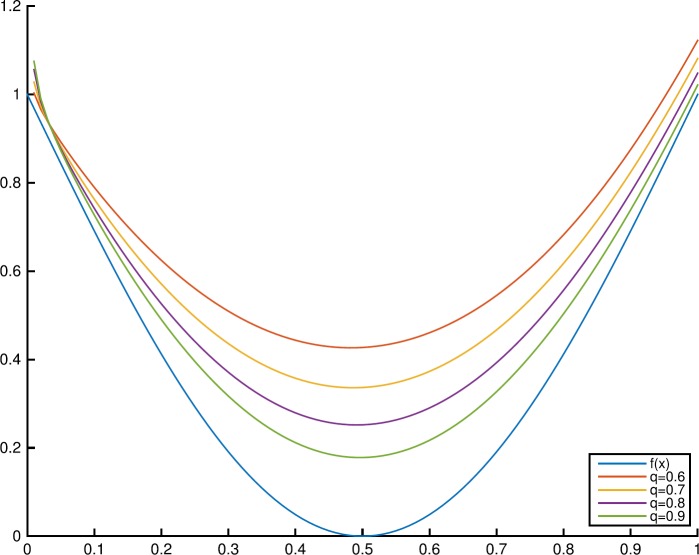
Monotonicity of $T_{n,q,\alpha }(f;x)$ in the parameter *q*

Letting the convex function $f(x) = 1 - \sin(\pi x)$, $x\in [0,1]$, the graphs of $f(x)$ and $T_{n,0.9,0.9}(f;x)$ with different values of $n=10, 15, 20, 30$ are shown in Fig. 3. Figure 4 shows the graphs of $f(x) = 1 - \sin(\pi x)$ and $T_{10,q,0.9}(f;x)$ with $q=0.6, 0.7, 0.8, 0.9$.

## Conclusion

In this paper, we proposed a new family of generalized Bernstein operators, named $(\alpha , q)$-Bernstein operators, and denoted by $T_{n,q,\alpha }(f)$. We study the rate of convergence of these operators, investigate their monotonicity-, convexity-preserving properties with respect to $f(x)$, and also obtain their monotonicity with *n* and *q* of $T_{n,q,\alpha }(f)$.
